# Variation in Oxygen Saturation by Pulse Oximetry During and After Breastfeeding Among Healthy Term Neonates During Early Postnatal Life at Tertiary Care Hospital

**DOI:** 10.7759/cureus.16564

**Published:** 2021-07-22

**Authors:** Sana Niaz, Vikram Kumar, Anum Rahim, Azeem Khan, Asma Bham, Syed Rehan Ali

**Affiliations:** 1 Neonatology, Indus Hospital & Health Network, Karachi, PAK; 2 Epidemiology and Public Health, Indus Hospital Research Center, Indus Hospital & Health Network, Karachi, PAK; 3 Indus Hospital Research Center, Indus Hospital & Health Network, Karachi, PAK

**Keywords:** breastfeeding, oxygen saturation, heart rate, postnatal life, suck swallow breathe coordination

## Abstract

Background

Breastfeeding plays a vital role in a newborn’s life as it increases its chances of survival and is considered the optimal nutritional source for newborns. All newborns must have developed the suck, swallow, and breathe coordination in order to safely breastfeed. Studies conducted on breastfeeding in healthy term babies are limited as most studies available on breastfeeding focus on preterm babies. Full-term healthy infants can also present with feeding difficulties but due to a lack of studies conducted on them, there is no existing oxygen saturation pattern for healthy term infants. Thus, our study is designed to observe variations in the oxygen saturation of healthy term infants during breastfeeding.

Methodology

A cross-sectional study was conducted in a tertiary care hospital from March 2021 to April 2021. Using a non-probability consecutive sampling technique, 60 neonates were enrolled in the study. The baby was monitored for heart rate and oxygen saturation before, during, and after feeding.

Results

The oxygen saturation levels were lower during feed while it was significantly high after a feed (p < 0.001). No significant variation was seen between saturation before feeding and during feed (0.635) or before feeding with after feed (p = 0.108). Maximum oxygen saturation drop was observed in 21% at the first minute and cumulatively 73% of neonates within the first five minutes of feeding. Heart rate remained in the physiological range (120-160 b/min) in 85%, above 160 in just 11.6% of the babies.

Conclusion

Effective breastfeeding is crucial for the growth and development of every infant, which is why there is a need to have an understanding of how infants develop suck, swallow, and breathe coordination. Having breathing and sucking patterns for infants can help medical personal identify when an infant is having difficulty with oral feeding and suggest safer, more effective methods of breastfeeding.

## Introduction

Breastfeeding is a vital part of every newborn’s life as it is considered to be an important and optimal source of nutrition for all newborns. Suck, swallow and breath coordination is optimal for safe and effective breastfeeding. Healthy term infants are able to simultaneously suck, swallow and breathe [1]. Vacuum is an important factor for the removal of milk from the breast. During sucking, when the tongue comes in opposition to the palate, it creates negative pressure, causing milk to flow in the oral cavity of the infant [2].

Oxygen saturation is a measure of how much hemoglobin is bound to oxygen versus how much hemoglobin remains unbound [3]. Desaturation is oxygen saturation less than 94% in babies >36 weeks postmenstrual age [4]. Saturation probe pulse oximetry is a non-invasive way to measure oxygen saturation and heart rate in blood. It also provides monitoring of cardiopulmonary changes during feeding [5]. The normal range of oxygen saturation measured by pulse oximetry is 95%-100% in preterm infants and 97%-100% in full-term infants and children [6]. A study conducted by Hammerman C and Kaplan M in Jerusalem, Israel included 21 infants where 11 were breastfed and ten were bottle-fed infants. The study did not report any variation in oxygen saturation during breastfeeding, however a drop in oxygen saturation to <90% was observed post breastfeeding [7].

Studies conducted on breastfeeding in healthy term babies are limited, as most studies on breastfeeding focus on preterm babies. Chen CH et al. conducted a prospective study in Taiwan on 25 babies for 80 feeding events with positive effects of breastfeeding, during which two episodes of apnea (holding of breath for more than 20 seconds) and 20 episodes of desaturation (PaO2 < 90%) were observed during bottle feeding and none during breastfeeding [8]. Suiter DM and Ruark-McMurtrey J conducted an observational study on healthy term neonates and infants at birth on the first week and second month of life, the study didn’t found variation in oxygen saturation and heart rate during and after feed [5].

Using existing studies, we know there is a sucking and breathing pattern of preterm and unwell infants during breastfeeding. Full-term healthy infants can also present with feeding difficulties but due to a lack of studies conducted on them, there is no existing oxygen saturation pattern for healthy term infants. Thus, our study is designed to observe variations in the oxygen saturation of healthy term infants during breastfeeding. Additionally, abnormal cardiopulmonary events during feeding may indicate that the infant is not tolerating breastfeeding and requires further swallowing assessment. Effective breastfeeding requires maturation and coordination of the suck, swallow and breathe reflex. Improving our understanding of the development of suck, swallow, and breathe coordination can help with the early identification of difficulties with oral feeding. This can help create assessment tools and early interventions for safe and effective breastfeeding.

The literature shows that there is a variability of oxygen saturation established with bottle feeding and preterm infants. The studies performed in term infant was conducted after the first week of birth or in infants, there was no study that observed the first two days of life, which is part of the fetus to neonatal transition and also, the closure of patent ductus arteriosus (PDA) and lung recruitment happens after birth [9]. The objective of this study is to determine the change in oxygen saturation of a term neonate during and after breastfeeding.

## Materials and methods

A cross-sectional study was conducted at the Well Baby Unit of Sheikh Saeed Memorial Campus of the Indus Hospital & Health Network from March 2021 to April 2021. Term and stable babies with age less than two days of life and baseline oxygen saturation of more than 94% were enrolled in the study. Babies who were born prematurely, with low birth weight, bottle feeding, sick or unstable neonates, mothers with inverted or flat nipples, and babies having any anomalies that can affect feeding and oxygen saturation like congenital heart disease were excluded. Babies were monitored with Medtronic Capnostream monitor (PM35MN, USA) with adhesive, clothed saturation probe.

Using a non-probability consecutive sampling technique, 60 neonates were enrolled in the study. Consent was taken from every mother prior to enrollment. Data was collected by the primary investigator and female research associate on predesigned proforma. The baby was monitored for heart rate and oxygen saturation one to two minutes before feeding and then for every minute for 10 minutes followed by one to two minutes after feeding.

Data was entered and analyzed using SPSS 26. Mean ± SD was reported for normally distributed continuous variables, that is, hours of life, gestational age, oxygen saturation, heart rate, and respiratory rate at birth. Frequency and percentages were reported for gravidity, parity, maternal comorbidities, gender, and mode of delivery type. The oxygen saturation at baseline, average O2 saturation during the feed and after the feed was to be compared using repeated measures ANOVA. Line graphs were made to see the variation in oxygen saturation during the course, that is, from baseline to after feed.

## Results

A total of 60 breastfeeding babies were enrolled in this study. Table 1 shows that the median maternal age was found to be 25 years. There were no known comorbidities among 71.7% of mothers, whereas gestational diabetes mellitus (GDM) was found in 25% of cases. Fifty-five percent of the neonates were born to primigravida mothers, while only 6.7% of women had gravida greater than 3. Mean gestational age was 38.5 ± 0.1 weeks. Thirty-two out of 60 neonates were born via spontaneous vaginal delivery while the rest were either delivered via emergency or elective cesarean section. About 43% of babies were male. Median birth weight of 2800 grams, length of 49 cm, and occipitofrontal circumference (OFC) of 34 cm. Data collection was done at the mean age of 16 hours of life.

**Table 1:** Demographic characteristics. EL LSCS: Elective lower (uterine) segment Caesarean section; EM LSCS: Emergency lower (uterine) segment Caesarean section; GDM: Gestational diabetes mellitus; HTN: Hypertension; SVD: Spontaneous vaginal delivery.

Demographic characteristics	
Variables	Frequency (%)
Maternal age	25 (22-29.8)
Hours of life	16.7 (±1.1)
Gestational age	38.5 (±0.1)
Birth weight	2.8 (2.6-3.1)
Length	49 (48-51)
OFC	34 (33-35)
Oxygen saturation at birth	97.1 (±0.1)
Temperature at birth	97 (36.4-37)
Heart rate at birth	143.2 (±0.9)
Respiratory rate at birth	47.5 (±0.5)
Gravida	
Primigravida	33 (55.0)
Gravida 2	16 (26.7)
Gravida 3	7 (11.7)
Gravida >3	4 (6.7)
Parity	
Para 1	19 (31.7)
Para 2	21 (35.0)
Para 3	16 (26.7)
Para >3	4 (6.7)
Maternal comorbidities	
None	43 (71.7)
GDM	15 (25.0)
HTN	1 (1.7)
Others	1 (1.7)
Mode of delivery	
SVD	32 (53.3)
EL LSCS	12 (20.0)
EM LSCS	16 (26.7)
Gender	
Male	26 (43.3)
Female	34 (56.7)

A repeated measures ANOVA with Greenhouse-Geisser correction was applied which determined that overall the oxygen saturation levels differed significantly (p = 0.002). Post hoc tests using the Bonferroni correction revealed that the oxygen saturation levels were lower during feed while it was significantly high after a feed (p < 0.001) (Table 2). However, no significant variation was seen between saturation before feeding and during feed (0.635) or before feeding with after feed (p = 0.108). Similarly, the heart rate was also analyzed, and it revealed that the overall variation was significantly present (p = 0.015). The heart rate before the feed was lower than that of during feed (p = 0.007) (Table 2). Additionally, there was no variation between the heart rate before and after a feed (p = 0.351) or during and after a feed (p = 0.376).

**Table 2:** Repeated measures ANOVA for oxygen saturation and heart rate.

	Before feed	During feed	After feed	P-value
Oxygen saturation	94.7 (3.6)	94.1 (2.1)	95.8 (2.8)	0.002
Heart rate	131.4 (14.9)	135.9 (12.4)	134.2 (13.6)	0.015

**Figure 1:**
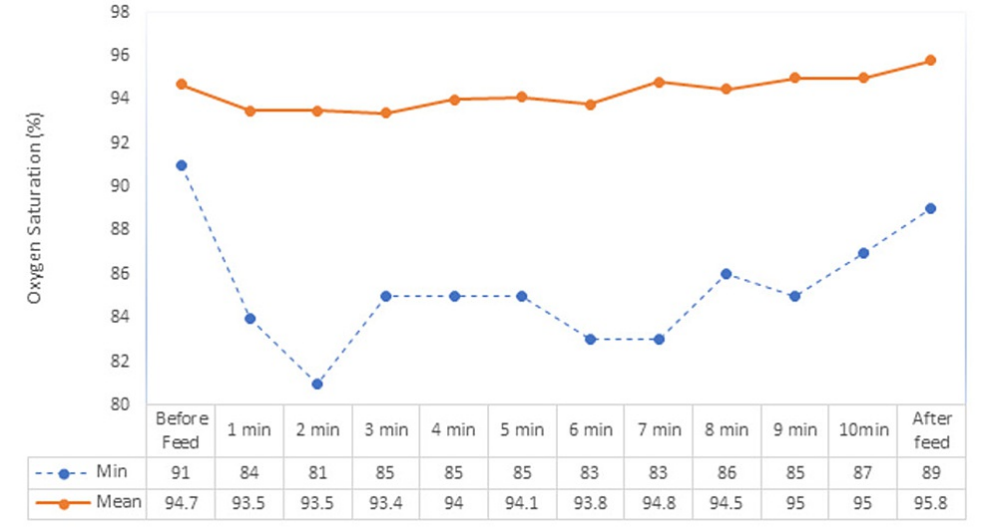
Oxygen saturation trend of neonates, before, during, and after feed.

Mean oxygen saturation before feeding was 94.7, as breastfeeding was started it dropped to 93.5, remained lower for the first six minutes from baseline, and regained baseline to 94.8 at seventh minute than remained above baseline that is within the physiological range. The oxygen saturation dropped between 90% and 94% in 35% and less than 90% in 41.6% of babies (Figure 1).

**Figure 2:**
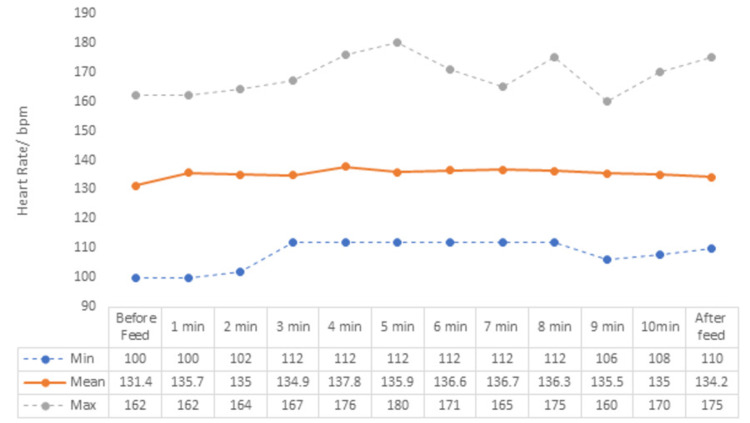
Heart rate trend of neonates, before, during, and after feed.

The mean heart rate was 131.4 beats per minute before feeding, which started to increase from the first minute and remained above baseline throughout the feeding (Figure 2). There was a maximum difference of 6.4 at the fourth minute of feeding and after feeding it remained higher with the difference of 2.8. All these ranges were in the physiological range but the heart rate dropped from the baseline in 15% of babies, the rise of less than 10 b/min was observed in 38.3% of babies, the rise of 10-30b/min was observed in 36.6%. A rise in heart rate was observed in the first five minutes in 46.6% and 36.6% in the next five minutes. Heart rate remained in the physiological range (120-160 b/min) in 85%, above 160 in just 11.6% of the babies.

## Discussion

Breastfeeding is an important part of newborn life. Preterm babies are able to suck more effectively from breastfeeding than bottle-feeding. It was also seen that bottle-feeding is associated with O2 desaturation in preterm babies along with bradycardia [4]. Swallowing mechanisms develop at 34 weeks of gestation so we assume that during feeding in term healthy babies, there will be no drop in oxygen saturation. However, Hammerman C and Kaplan M did a study in term healthy infants during breast and bottle feeding and they did not find any variation during breastfeeding while they did find a significant drop in oxygen saturation post-feeding [7]. Suiter DM and Ruark-McMurtrey J conducted an observational study on healthy term neonates and infants at birth in the first week and the second month of life, the study did not find variation in oxygen saturation and heart rate [5]. We conducted this study on 60 babies during breastfeeding and found that there is a significant drop in oxygen saturation during feeding to 84% and heart rate increase during breastfeeding compared to before the start of the feed.

During fetal life, ductus arteriosus which connects the pulmonary artery to the arch of the aorta as blood flow bypasses the lung and the placenta plays an important role. After birth, the placenta is removed and the lungs start to function, leading to the closure of ductus arteriosus due to increase oxygen concentration and decrease prostaglandins. These changes occur during the first two days of life. The physiological impact of breastfeeding is not well studied in term healthy babies especially within the first two days of life, which is a period of PDA closure and lung recruitment.

We used pulse oximetry to monitor oxygen saturation and heart rate during breastfeeding. Pulse oximetry is a non-invasive way to measure oxygen saturation and heart rate. It provides monitoring of cardiopulmonary changes during feeding [5]. Some feeding events lasted longer than 10 minutes but we did not interrupt the natural process. While some babies took feed only for 10 minutes thus, the measurement was taken for that period. We found a significant drop in oxygen saturation, up to 84% during feeding, while saturation increased during the post-feeding period. Similarly, we found an increase in heart rate during feeding, which may be due to the activation of the sympathetic nervous system.

Competent breastfeeding is seen in the majority of term babies, only 25% to 45% of babies may experience feeding difficulties [10]. Management of oral feeding difficulties still lacks evidence-based support [11]. Effective breastfeeding requires maturation and coordination of the suck, swallow and breathe reflex. Improving our understanding of how suck, swallow, breath coordination develops can help us identify oral feeding difficulties earlier. This will help us develop assessment tools and early interventions for safe and effective breastfeeding. Moreover, with a better understanding of these physiological functions, every baby will be tailored on functional maturity level rather than on their gestational age.

Like all studies, our study has some potential limitations. This was a single-center study conducted at one tertiary care hospital located in the largest city in Pakistan. The results from the sample may not be generalized to the larger population. Data for this study was collected from only 60 infants. This is not sufficient to be representative of the population. In addition to the small sample size, no comparison group was present. However, our study was one of the first of its kind to be conducted in the Pakistani setting. This study has highlighted the need for a larger randomized control trial, which may help us to find normal and abnormal cardiopulmonary responses that occur during feeding. Cardiopulmonary events must be assessed for any abnormalities that can indicate when the baby is not tolerating feed and needs to be evaluated for dysfunctional swallowing. There is also a need to further study the effect of changing the position of the baby during feed on oxygen saturation. Furthermore, there is a need for evaluating neurocognitive function in infants in the long term.

## Conclusions

The study observed differences between the oxygen saturation levels in infants during and after a feed. Infants had higher oxygen saturation after a feed. There was no difference observed in the heart rate before and after a feed but an increase in heart rate was observed during a feed. Effective breastfeeding is crucial for the growth and development of every infant, which is why there is a need to have an understanding of how infants develop suck, swallow and breathe coordination. Having breathing and sucking patterns for infants can help medical personnel identify when an infant is having difficulty with oral feeding and suggest safer and more effective methods of breastfeeding.
